# Incidence and survival rate of women with cervical cancer in the Greater Amsterdam area

**DOI:** 10.1038/sj.bjc.6601157

**Published:** 2003-08-26

**Authors:** S Bulk, O Visser, L Rozendaal, R H M Verheijen, C J L M Meijer

**Affiliations:** 1 Department of Pathology, VU University Medical Center, PO Box 7057, 1007 MB Amsterdam, The Netherlands; 2 Comprehensive Cancer Centre Amsterdam (Integraal Kankercentrum Amsterdam), PO Box 9236, 1006 AE Amsterdam, The Netherlands; 3 Department of Obstetrics & Gynaecology, VU University Medical Center, PO Box 7057, 1007 MB Amsterdam, The Netherlands

**Keywords:** cervical cancer, incidence, survival, adenocarcinoma, squamous cell carcinoma

## Abstract

To evaluate the effect of population-based cervical cancer screening on the occurrence of cervical cancer in The Netherlands, we investigated the incidence and survival of cervical cancer registered by a cancer registry in the Greater Amsterdam area. The incidence rate of squamous cell carcinoma decreased significantly from 9.2/100 000 women in 1988 to 5.9/100 000 in 2000 (*P*<0.001). The incidence rate of adenocarcinomas remained stable. After adjustment for age, stage and lymph node involvement, the relative risk of death was 1.6 times higher for patients with adenocarcinomas than for patients with squamous cell carcinoma (95% CI 1.2–2.1). The decreased survival was related to histological type, as the effect remained significant after correction for confounding factors. Over time, the prognosis of women with squamous cell carcinoma improved significantly. No significant change was observed for women diagnosed with adenocarcinoma. These results suggest that the screening programme in The Netherlands as executed in the Greater Amsterdam area is associated with a decreased incidence and increased survival of patients with squamous cell carcinoma, but fails to detect (pre)malignant lesions of adenocarcinoma. Since more than 92% of adenocarcinomas and its precursors contain high-risk HPV, adding HPV testing to cytologic screening might improve the present screening programme in detecting adenocarcinoma and its precursor lesions.

Population-based cervical cancer screening has led to a decrease in the incidence of cervical cancer (Gustafsson *et al*, 1997; Vizcaino *et al*, 2000). However, recent data suggest that the decrease in incidence is caused by a decrease of squamous cell carcinoma, while the incidence of adenocarcinoma of the cervix shows no change or sometimes even an increase (Anttila *et al*, 1999; Bergstrom *et al*, 1999; Hemminki *et al*, 2001; Liu *et al*, 2001).

It has been suggested that the unchanged or even increased incidence of adenocarcinoma of the cervix is the result of systematic underscreening of cervical smears for (pre)malignant changes of adenocarcinoma of the cervix (Parkin *et al*, 1985; Mitchell *et al*, 1995; Stockton *et al*, 1997). Moreover, patients with adenocarcinomas of the cervix are considered to have a decreased survival compared to patients with squamous cell carcinomas (Hopkins and Morley, 1991; Lai *et al*, 1999; Nakanishi *et al*, 2000; Grisaru *et al*, 2001). It has been suggested that this decreased survival is associated with higher stages of disease with which patients with adenocarcinomas are detected.

Here, we report on the incidence and survival rates of cervical cancer cases registered by a cancer registry in a large geographically defined region of The Netherlands, the Greater Amsterdam area. Special attention was paid to trends in incidence and survival rates for cases of squamous cell carcinoma and adenocarcinoma.

## MATERIALS AND METHODS

### Data collection

The cancer registry of the Comprehensive Cancer Centre Amsterdam (CCCA, ‘Amsterdam Cancer Registry’) is a population-based cancer registry since 1988, and part of the nationwide Netherlands Cancer Registry as of 1989. It covers two out of 12 Dutch provinces: Noord-Holland and the major part of Flevoland. The population of the CCCA region increased from 2.50 million on 1 January, 1988 to 2.80 million on 1 January, 2001. All malignant tumours were registered in all 20 hospitals in the region, comprising two university hospitals and a specialised cancer hospital (where cancer patients are treated).

Clinical information and pathology data were extracted from the medical records. Apart from demographic data, data were collected on tumour site, morphological classification (according to the International Classification of Diseases for Oncology) and stage of the tumour. The fourth edition of the TNM-classification was used whenever applicable (Hermanek and Sobon, 1987). In 1988–1993, the FIGO stage was registered separately, and after 1993 the FIGO stage was derived from TNM stage. Data concerning participation in cervical cancer screening programmes were not available.

For this study, all cervical cancer cases diagnosed between 1 January, 1988 and 31 December, 2000 were selected from the cancer registry. The following tumours were excluded: noninvasive tumours and tumours diagnosed in patients living outside the CCCA region. Patients diagnosed in a hospital outside the region, but living in the CCCA region were included. Information on the vital status of all patients was collected in the hospitals and from general practitioners. However, the majority of the information on vital status was obtained from record linkage of computerised data on all deceased persons in the study period that were made available by 51 of the 74 municipalities in the CCCA region (covering more than 85% of the population). Inquiries about the vital status of patients living in the other 23 municipalities were made at the municipal population registers and at the Central Office for Genealogy of The Netherlands, The Hague. Less than 1% of the cases were lost to follow-up. In the survival analyses, cases diagnosed in 1998–2000 were excluded, because of the short period of follow-up. Tumours first diagnosed at autopsy, second (or third, etc.) tumours and noncarcinomas were also excluded from the survival analyses. Follow-up of the patients diagnosed in 1988–1997 was complete until at least 1 January, 1999.

Population data of The Netherlands were obtained from Statistics Netherlands (CBS, Voorburg/Heerlen, The Netherlands). Data from Statistics of Netherlands were also obtained with respect to the survival of the general Dutch population.

### Statistical analysis

Incidence of cervical cancer was calculated per 100 000 person years. Direct standardisation was used for age adjustment with respect to the European standard population, and the European standardised rates (ESRs) were calculated. Trends in the incidence of the ESR were investigated by calculating the estimated annual percent change (EAPC) (Visser *et al*, 2001). For the analyses, cases were divided into squamous cell carcinoma, adenocarcinoma including adenosquamous carcinoma and other histological type (i.e., other and unspecified carcinomas, sarcomas and undefined tumours). Age was divided into 15-year categories. However, age categories of 15–29 and 30–44 years were analysed jointly in the survival analyses, based on the low number of cases (*n*=90) in the category of 15–29 years. Differences in distribution over stage and age categories were assessed with *χ*^2^ statistics.

Relative survival and 95% confidence intervals (CIs) were calculated as a measure of disease-specific survival (Hakulinen and Abeywickrama, 1985). The relative survival is the ratio between crude and expected survival and is close to disease-specific survival. We did not calculate disease-specific survival, because the cause of death was not available as linkage with the death registry in The Netherlands is not possible.

The Cox multivariate regression analysis for survival was performed to investigate survival. Associations were examined for all cases, and for cases of squamous cell carcinoma and adenocarcinoma separately. In the analyses, the FIGO classification was used to adjust for stage. Lymph node status is not considered in the FIGO classification for cervical carcinoma, and therefore it was introduced in the analyses as a separate variable. For cases diagnosed in 1988, TNM was not registered, so these cases were classified as ‘nodal involvement unknown’. Age, stage and nodal involvement were divided into categories and entered into the model as dummy variables. *P*-values of 0.05 or less were considered statistically significant. Using STATA 6.0 for Windows, hazard ratios (HRs) and 95% CIs were calculated.

## RESULTS

From 1988 up to 2000, 1925 patients were diagnosed with invasive cervical cancer (Table 1

**Table 1:** Incidence of cervical cancer in the Greater Amsterdam area, the Netherlands, 1988–2000

	**SCC**		**AdCx**		**Other**		**Total**	
**Year**	**Cases**	**ESR**	**Cases**	**ESR**	**Cases**	**ESR**	**Cases**	**ESR**
1988	128	9.7	22	1.6	7	0.5	157	11.8
1989	129	9.5	24	1.8	2	0.2	155	11.5
1990	125	8.8	29	2.2	4	0.3	158	11.3
1991	111	7.6	19	1.2	4	0.3	134	9.0
1992	134	9.5	28	1.9	6	0.4	168	11.9
1993	126	8.9	30	2.1	2	0.2	158	11.1
1994	114	7.5	26	1.8	4	0.3	144	9.6
1995	120	7.8	25	1.7	3	0.2	148	9.7
1996	107	6.8	18	1.2	7	0.4	132	8.5
1997	117	7.4	32	2.1	4	0.2	153	9.6
1998	118	7.5	33	2.1	2	0.1	153	9.6
1999	108	6.8	17	1.1	5	0.3	130	8.2
2000	100	6.1	26	1.6	9	0.6	135	8.2

EAPC		−3.2%		−1.1%		−1.4%		−2.7%
*P*-value		<0.001		0.54		0.74		0.001

SCC=squamous cell carcinoma; AdCx=adeno(squamous)carcinoma; ESR=European standardised rate; EAPC= estimated annual percent change.

). The annual number of incident cases of cervical cancer decreased from 157 patients registered in 1988 to 135 patients in 2000. The ESR decreased from 11.8/100 000 women in 1988 to 8.2/100 000 women in 2000. The total ESR decreased by 2.7% annually (*P*=0.001). This decrease in incidence was mainly caused by a decrease in the incidence of squamous cell carcinoma cases, as the EAPC in ESR for squamous cell carcinomas was −3.2% (*P*<0.001). For adenocarcinomas, there was no statistically significant trend in the incidence (EAPC −1.1%, *P*=0.54). The incidence of other cervical malignancies decreased with 1.4% annually (*P*=0.74). During the study period, the contribution of adenocarcinomas to the total number of malignancies increased from 16% in 1988–1990 to 18% in 1998–2000.

**Figure 1:**
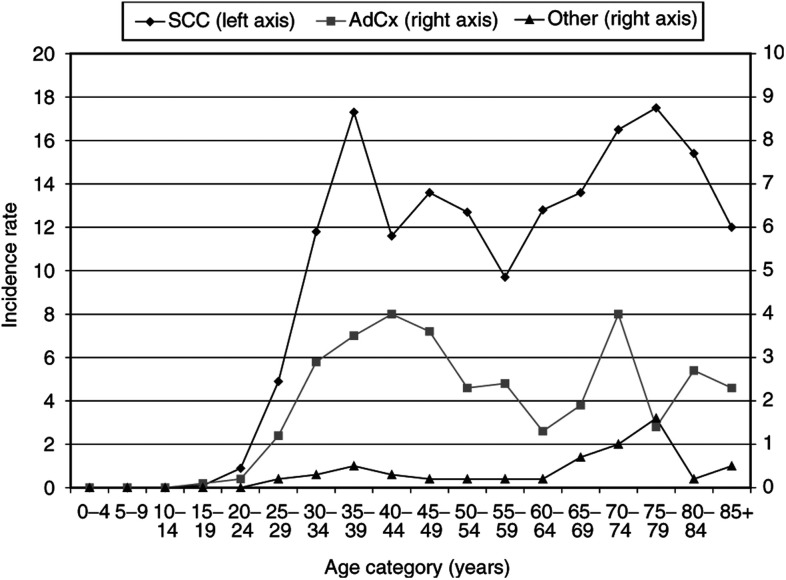
Age-specific incidence rates by histologic type of patients in the Greater Amsterdam area 1988–2000.

The median age of patients with adenocarcinoma was 3 years below the median age of patients with squamous cell carcinoma, that is 44 years (range: 18–92) and 47 years (range: 19–98), respectively (*P*<0.05). Age-specific incidence was highest in age groups 35–39 and 70–84 years for squamous cell carcinoma patients, and the age-specific incidence was highest in the age group 35–49 years for adenocarcinoma patients (Figure 1

).

**Figure 2:**
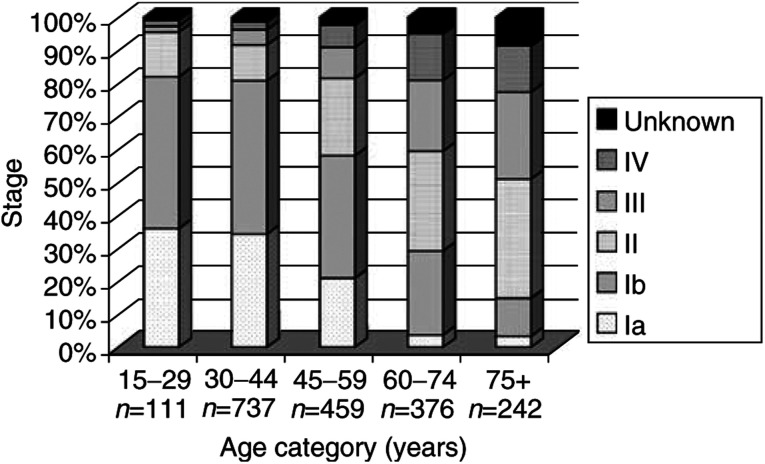
Stage at diagnosis by 15-year age category of patients in the Greater Amsterdam area, the Netherlands, 1988–2000.

**Figure 3:**
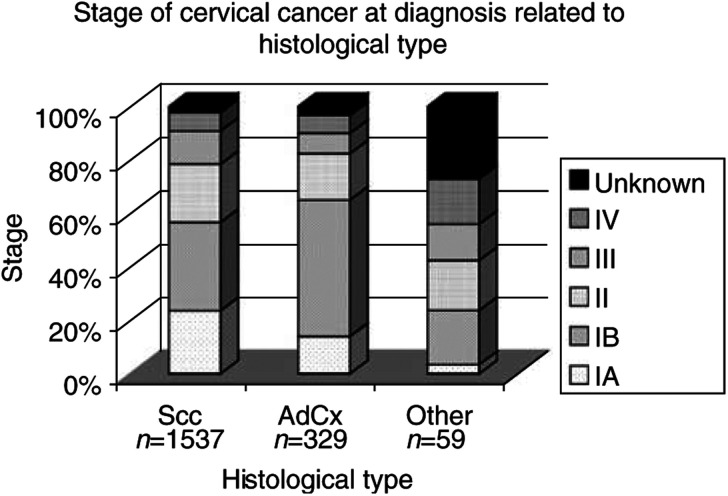
Stage at diagnosis by histologic type of patients in the Greater Amsterdam area, the Netherlands, 1988–2000.

Younger patients were more often diagnosed in early stages of cancer than older patients (Figure 2

). Of women 15–29 years, 82% were diagnosed with FIGO stage I disease, while only 15% of patients 75 years and over were diagnosed with FIGO stage I disease. Only 2% of the patients in the age category 15–29 years were diagnosed in stage IV, while 14% of the oldest patients were diagnosed in this stage. Figure 3

shows that 55% of squamous cell carcinomas and 65% of patients with adenocarcinomas were diagnosed in FIGO stage I (*P*=0.003). However, adenocarcinomas were diagnosed less often in the microinvasive (i.e., Ia) stage of the disease than squamous cell carcinomas (15 and 22%, respectively) (*P*<0.0001). The percentage of cases diagnosed in FIGO stage IV did not differ statistically significantly between squamous cell carcinomas (7%) and adenocarcinomas (6%) (*P*=0.71). During the study period, there were no statistically significant changes in distribution over the stages.

During 1988–1997, there were 1441 patients with cervical carcinoma in the region of the CCCA, 480 of whom died (33.3%). The median follow-up time was 56 months (range: 0–165 months). The overall 5-year relative survival of cervical carcinoma was 71% (Table 2

**Table 2:** Relative survival of patients with cervical carcinoma in the Greater Amsterdam area, the Netherlands in 1988–1997

**Variable**	**Category**	**Cases**	**5-year survival (%)**	**95% CI (%)**
All cases		1441	71	68–74

Age	<45	665	85	82–88
	45–59	321	72	66–77
	60–74	285	54	47–60
	75+	170	41	31–52

FIGO stage	I	831	91	88–93
	II	304	57	50–64
	III	168	35	27–43
	IV	99	16	9–24
	Unknown	39	44	27–61

Histology	SCC	1169	72	69–75
	AdCx	244	66	59–72
	Other/unspecified carcinoma	28	60	38–78

CI=confidence interval; SCC=squamous cell carcinoma; AdCx=adeno(squamous)carcinoma.

). Relative survival decreased from 85% for women <45 years to 41% for women of 75 years and over. Patients with FIGO stage I had a relative survival of 91% decreasing to 16% for tumours diagnosed in FIGO stage IV. Relative survival of squamous cell carcinomas was 72% (95% CI: 69–75%), and somewhat lower for adeno(squamous)carcinomas (66%, 95% CI: 59–72%).

Table 3

**Table 3:** Relative risk of death for patients in the Greater Amsterdam area, The Netherlands, with cervix carcinoma diagnosed in 1988–1997 (*n*=1441)

	**Univariate**			**Multivariate^a^**	
**Factor**	**Cases**	**Hazard ratio**	**95% CI**	**Hazard ratio**	**95% CI**
FIGO stage					
I	831	1	Reference	1	Reference
II	304	4.9*	3.8–6.3	3.3*	2.6–4.7
III	168	10*	7.9–13	6.4*	4.7–8.5
IV	99	19*	14–25	11*	7.6–15
Unknown	39	9.2*	5.9–14	5.7*	3.6–9.1

Nodal involvement					
No	563	1	Reference	1	Reference
Yes	668	3.2*	2.5–4.1	2.1*	1.6–2.7
Unknown^b^	210	1.6*	1.3–2.0	1.4*	1.1–1.8

Morphological type					
SCC	1169	1	Reference	1	Reference
AdCx	244	1.1	0.9–1.4	1.6*	1.3–2.1
Other/unspec. carcinoma	28	1.7	1.0–3.0	0.7	0.4–1.2

Year of diagnosis					
1988/1990	451	1	Reference	1	Reference
1991/1993	442	0.7*	0.6–0.9	0.8*	0.6–1.0
1994/1997	548	0.8	0.7–1.1	0.8	0.6–1.0

a Adjusted for age category and all other factors in the table.

b Cases diagnosed in 1988 were all classified as unknown.

* *P*<0.05.CI=confidence interval; SCC=squamous cell carcinoma; AdCx=adeno(squamous)carcinoma.

displays the results of the multivariate analysis of survival for all types of cervical cancer. Compared to FIGO stage I, all other stages had significantly increased HRs. Lymph node status is not considered in the FIGO classification for cervical carcinoma. For cases diagnosed in 1988, TNM was not registered and these cases were classified as ‘nodal involvement unknown’. However, nodal involvement appeared to be associated with an increased risk of death (HR 2.1, 95% CI 1.6–2.7). Tumour histology was investigated with squamous cell carcinoma cases as reference category. Univariately, the HR of adenocarcinomas was slightly increased (HR 1.1), while other/unspecified carcinomas were associated with a significant increase in risk. The increased risk for other/unspecified carcinomas disappeared with multivariate adjustment, indicating that the increase in risk was caused by confounding by age and stage. After adjustment, the HR for adenocarcinomas was significantly increased (HR 1.6, 95% CI 1.3–2.1). Over time, the prognosis of patients with cervical cancer improved, as the multivariate HRs were decreased for the periods 1991–1993 and 1994–1997 as compared to the reference period 1988–1990. Adjustment for stages 1a and 1b separately did not alter these findings substantially (data not shown).

Squamous cell carcinoma cases and adenocarcinoma cases were also analysed separately (Table 4

**Table 4:** Relative risk of death for patients in the Greater Amsterdam area, The Netherlands, with cervix carcinoma diagnosed in 1988–1997 (*n*=1441). Year of diagnosis as an indicator of survival, separately for patients with squamous cell carcinoma and adenocarcinoma

		**Univariate**		**Multivariate^a^**	
**Factor**		**Hazard ratio**	**95% CI**	**Hazard ratio**	**95% CI**
Year of	1988/1990	1	Reference	1	Reference
diagnosis;	1991/1993	0.9	0.5–1.5	0.8	0.5–1.5
AdCx	1994/1997	1.0	0.6–1.6	1.2	0.7–2.0

Year of	1988/1990	1	Reference	1	Reference
diagnosis;	1991/1993	0.7*	0.6–0.9	0.8*	0.6–1.0
SCC	1994/1997	0.8	0.6–1.0	0.8*	0.6–1.0

a Adjusted for age, stage and nodal involvement.

* *P*<0.05.CI=confidence interval; AdCx=adeno(squamous)carcinoma; SCC=squamous cell carcinoma.

). Both for squamous cell carcinomas and adenocarcinomas, survival decreased with increasing age, higher FIGO stage and positive lymph nodes at diagnosis (data not shown). The improvement in survival of women with cervical cancer during the period 1988–1997 was associated with cases of squamous cell carcinoma only. Survival of women with cervical adenocarcinoma did not improve during the study period (Table 4).

## DISCUSSION

Our results show that the incidence of cervical cancer has decreased significantly during the period 1988–2000, and that this decrease is caused by a decrease in the incidence of squamous cell carcinomas. In multivariate analyses, survival for patients diagnosed with adenocarcinoma of the cervix was significantly lower than survival for patients with squamous cell carcinoma. This indicates that women with adenocarcinoma of the uterine cervix have an intrinsically increased risk of death compared with women with squamous cell carcinoma independent of stage, age and nodal involvement.

We studied patients with cervical cancer who were all diagnosed within a geographically defined region in The Netherlands: the Greater Amsterdam area. As data were obtained by the Regional Cancer Registry, we were able to study an unbiased population of women with different histological types of cervical cancer for factors associated with survival. In this study, the histological verification rate was 99.8%. Nationally, the histological verification rate for cervical cancer is 99.7%, indicating a high accuracy rate of the Dutch Cancer Registries (Visser *et al*, 1997).

In this study, knowledge of participation in cervical cancer screening preceding the diagnosis of cancer may have been relevant. We did not have data on either individual Pap smear taking or participation in the nationwide screening programme in this group of women with cervical carcinoma. Cytological screening on an individual basis has been available for women in this region of The Netherlands since the 1970s. A nationwide screening programme aimed at specific age categories was initiated in 1988. Between 1988 and 1996, women aged 34–54 years were screened triannually, and from 1996 onwards, women aged 30–60 years are screened every 5 years. Overall, the coverage of cervical cancer screening activities over a period of 5 years is approximately 80% (van Ballegooijen and Hermens, 2000). However, regional participation in each screening round of the population-based programme is lower (60–70%). Even without data on screening participation, some findings do suggest the efficacy of screening in this region of The Netherlands. The incidence of cervical squamous cell carcinoma decreased significantly, while no statistically significant change in the incidence of adenocarcinoma was found. Previous studies have reported that cervical adenocarcinoma and its preinvasive stages are diagnosed less efficiently by Pap smear screening than squamous cell lesions (Mitchell *et al*, 1995; Stockton *et al*, 1997). In some countries, not only the absence of a decrease in incidence (Sigurdsson, 1993; Nieminen *et al*, 1995), but also increases in the incidence of cervical adenocarcinoma have been described in the presence of a screening programme (Bergstrom *et al*, 1999; Hemminki *et al*, 2001; Liu *et al*, 2001). One study even suggested an increase in incidence especially in younger women (Anttila *et al*, 1999), whereas older women were not affected by increases in the incidence rate of cervical cancer. Our data do not support this trend. Our findings suggest a positive effect of the screening programme as shown by the decrease in cervical carcinoma incidence. Increasing screening participation to obtain an even higher degree of coverage will most likely increase the efficacy of the screening programme in decreasing the incidence of cervical cancer.

In our analyses, patients with cervical adenocarcinoma had a worse prognosis than patients with squamous cell carcinoma after correction for confounders such as age, stage and nodal involvement. Previous studies either usually lacked sufficient numbers of patients with adenocarcinomas (Pilch *et al*, 2001), or were not population-based (Hopkins and Morley, 1991; Nakanishi *et al*, 2000), or did not make direct comparisons between patients with squamous cell carcinoma and adenocarcinoma (Benedet *et al*, 1998). Some studies attributed the decreased survival of women with adenocarcinoma to differences in histological type (Lai *et al*, 1999; Grisaru *et al*, 2001). In our study, patients with adenocarcinoma presented with more advanced stage I tumours than patients with squamous cell carcinomas. Still, patients with cervical adenocarcinoma were slightly younger at diagnosis than patients with squamous cell carcinoma, 48 and 50 years, respectively. As the association between adenocarcinomas and a worse prognosis increased after correction for confounding factors, this indicates that women with cervical adenocarcinoma have an inherently worse prognosis than women with squamous cell carcinoma. The main causal factor for the development of both squamous cell carcinoma and adenocarcinoma of the uterine cervix is infection with high-risk types of the human papillomavirus (hrHPV) (Walboomers *et al*, 1999; Bosch *et al*, 2002). There are substantial differences with respect to the exact type of hrHPV and the histological diagnosis. Adenocarcinomas are more often associated with HPV type 18 than squamous cell carcinomas (Bosch *et al*, 1995, 2002; Andersson *et al*, 2001; Clifford *et al*, 2003). Moreover, HPV 18 has been shown to be associated with a worse prognosis than other HPV types (Hildesheim *et al*, 1999; Schwartz *et al*, 2001), but this finding has been challenged (Pilch *et al*, 2001). An intrinsic difference between neoplasically converted squamous and cylindrical epithelium might also be the cause of the difference in prognosis, independent of HPV infection.

The survival of patients with squamous cell carcinoma increased during 1988–1997, while the survival of patients with adenocarcinomas did not change significantly. During the study period, there were no large changes in treatment of patients with cervical cancer in any stage of the disease. The nationwide screening programme was introduced in 1988. Theoretically, an improvement in the prognosis of patients with squamous cell carcinomas might have been caused by a major change in stage at diagnosis during the study period. We did not observe such a change in stage at diagnosis over time (data not shown). However, we cannot exclude an effect of subtle changes within broad stages, such as a shift from FIGO 1b to FIGO 1a. Screening, leading to an earlier detection of cervical cancer even without significant changes in broad FIGO stage distribution, may contribute to such an effect. We were not able to find a trend over time in histological lymph node positivity over all stages of disease, as lymph node sampling is dependent on stage at diagnosis. In this study, in 31% of women with adenocarcinoma and 43% of women with squamous cell carcinoma, lymph node status was not determined histologically. Stage migration due to improved staging procedures might also have contributed to the increased survival. Still, the statistically significant difference in survival was based on small numbers of adenocarcinoma cases compared to squamous cell carcinoma cases. Therefore, caution should be taken into account when interpreting trends in the survival of adenocarcinoma based on these data.

The overall 5-year survival rate was 71%. Compared to other countries in Europe, this is a high survival rate, with only the Nordic countries having comparable survival rates (Berrino *et al*, 1999). Other studies have also shown that within Europe, The Netherlands have a very low incidence, and a very high survival compared with other countries (Levi *et al*, 2000). It is reasonable to attribute these findings to the presence of a nationwide screening programme, as similar effects on incidence have been described in other countries with a screening programme (Gustafsson *et al*, 1997). Whether the effect on survival in the absence of changes in treatment can be exclusively attributed to the cervical cancer screening programme, remains a question of debate (Quinn *et al*, 1999; Sasieni and Adams, 1999).

Our study suggests that the present screening programme for cervical cancer is efficient in detecting (pre) malignant stages of squamous cell carcinoma, but fails to detect (pre) malignant stages of adenocarcinoma (Sasieni and Adams, 2001). Since more than 92% of the adenocarcinomas and its precursors contain hrHPV (Pirog *et al*, 2000; Pilch *et al*, 2001), adding hrHPV testing to conventional cytological screening might improve the present screening programme in detecting adenocarcinoma and its precursor lesions. This should be a focus of further research concerning the functioning of the screening programme for cervical cancer.

In conclusion, in the Greater Amsterdam area, the incidence of squamous cell carcinomas has decreased while there were no changes in the incidence of adenocarcinoma of the uterine cervix. Cases of adenocarcinoma of the uterine cervix are associated with a decreased survival rate compared to patients with squamous cell carcinoma. This decreased survival is related to tumour histology itself, since after correction for factors such as age, stage and lymph node status, the survival of adenocarcinoma patients is still lower compared with squamous cell carcinoma patients.
